# The enemy in the mirror: self-perception-induced stress results in dissociation of psychological and physiological responses in patients with dissociative disorder

**DOI:** 10.1080/20008198.2018.1472991

**Published:** 2018-06-18

**Authors:** Eva Schäflein, Heribert Sattel, Ulrike Schmidt, Martin Sack

**Affiliations:** a Department of Psychosomatic Medicine and Psychotherapy, Klinikum rechts der Isar, Technical University of Munich, Munich, Germany; b Department of Psychiatry and Psychotherapy, University Medical Centre of Göttingen, RG Stressmodulation of Neurodegeneration, Göttingen, Germany

**Keywords:** Autonomic nervous system, avoidance, dissociative disorder, face in the mirror, impedance cardiography, mirror-confrontation, parasympathetic, post-traumatic stress disorder, self-perception, sympathetic, sistema nervioso autónomo, evitación, trastorno disociativo, mirarse en el espejo, cardiografía de impedancia, espejo-confrontación, sistema nervioso parasimpático, trastorno de estrés postraumático, autopercepción, sistema nervioso simpático, 自主神经系统回避, 分离障碍, 面对镜子, 阻抗心动描记法, 镜面对抗, 副交感神经, 创伤后应激障碍, 自我认知, 同情心, • Dissociative disorder (DD) patients conducted facial mirror exposure.• This resulted in increased subjective stress and acute dissociation.• In contrast, impedance cardiography showed autonomic blunting.• Self-perception is potentially an important target for psychotherapy in DD.

## Abstract

**Background**: Patients suffering from dissociative disorders (DD) are characterized by an avoidance of aversive stimuli. Clinical experience has shown that DD patients typically avoid the confrontation with their own faces in a mirror (CFM).

**Objective**: To investigate potential CFM-associated self-reported and psychophysiological stress reactions of DD patients, which most likely inform on the still unknown pathophysiology of dysfunctional self-perception in DD.

**Method**: Eighteen DD patients and 18 healthy controls (HCs) underwent CFM. They were assessed for CFM-induced subjective self-reported stress, acute dissociative symptoms and sympathetic and parasympathetic drive using impedance cardiography.

**Results**: DD patients experienced more subjective stress and acute dissociation than HCs upon CFM. Their psychological stress response did not activate the sympathetic and parasympathetic nervous system.

**Conclusions**: In DD patients, CFM constitutes serious self-reported stress and is associated with a blunted autonomic reactivity. Therapeutic approaches promoting self-perception and self-compassion, in particular by using CFM, might serve as goal-oriented diagnostic and therapeutic tools in DD.

## Abstract (es)

**Antecedentes**: Los pacientes que sufren trastornos disociativos (DD en su sigla en inglés) se caracterizan por evitar los estímulos aversivos. La experiencia clínica ha mostrado que los pacientes con DD típicamente evitan la confrontación con sus propias caras en un espejo (CFM en su sigla en inglés).

**Objetivo**: Investigar las potenciales reacciones de estrés psicofisiológicas y autoinformadas asociadas a CFM de pacientes con DD, las que muy probablemente informan sobre la fisiopatología todavía desconocida de la autopercepción disfuncional en los DD.

**Método**: Dieciocho pacientes con DD y 18 controles sanos (HCs en sus siglas en inglés) se sometieron a CFM. Fueron evaluados en cuanto al estrés subjetivo auto-informado inducido por CFM, síntomas disociativos agudos e impulso simpático y parasimpático, usando cardiografía de impedancia.

**Resultados**: Los pacientes con DD experimentaron más estrés subjetivo y disociación aguda que los HCs en CFM. Su respuesta de estrés psicológico no activó el sistema nervioso simpático y parasimpático.

**Conclusiones**: En pacientes con DD, el CFM constituye un estrés grave auto-informado y se asocia con una reactividad autonómica limitada. Los enfoques terapéuticos que promueven la autopercepción y la autocompasión, en particular mediante el uso de CFM, podrían servir como herramientas diagnósticas y terapéuticas orientadas a objetivos en los DD.

## Abstract (zh)

**背景**：患有分离性障碍（DD）的患者的特征是回避厌恶性刺激。 临床经验表明，DD患者通常回避面对他们自己的镜像（CFM）。

**目的**：探讨DD患者潜在的CFM相关的自我报告和心理生理应激反应，这些反应很可能提示了还未被了解的DD的自我知觉病理生理功能障碍。

**方法**：18名DD患者和18名健康对照（HCs）进行CFM。 采用主观自我报告评估CFM诱导的压力和急性分离症状，使用阻抗心动描记法评估交感神经和副交感神经驱动。

**结果**：DD患者在CFM时比HC感受到更多的主观压力和急性分离。 他们的心理压力反应没有激活交感神经和副交感神经系统。

**结论**：在DD患者中，CFM构成严重的自我报告压力，并且与迟钝的自主神经反应相关。 促进自我认知和自我同情的治疗方法，尤其是通过使用CFM，可以作为DD治疗中以目标为导向的诊断和治疗工具。

## 1. Introduction

### 1.1. Dissociation and dissociative disorders

According to the Fifth Edition of the Diagnostic and Statistical Manual of Mental Disorders (DSM-5), Dissociative Disorders (DD) are characterized by a ‘disruption of and/or discontinuity in the normal integration of consciousness, memory, identity, emotion, perception, body representation, motor control, and behavior’ (American Psychiatric Association, 2013, p. 291). The question of what can be defined as dissociation is a topic of ongoing debate even among experts (Dell, 2011; Holmes et al., 2005; Nijenhuis & van der Hart, 2011). Dissociative symptoms are very prevalent in patients with common mental disorders, e.g. in Borderline Personality Disorder, Depersonalization-Derealization Disorder, or Post-traumatic Stress Disorder (PTSD), and are thus clinically relevant (Sar, 2011). DD are often underdiagnosed (Ginzburg, Somer, Tamarkin, & Kramer, 2010; Leonard, Brann, & Tiller, 2005; Spiegel, 2006; Wirtz & Frommberger, 2013) and have been associated with a poor psychotherapy outcome (Kleindienst et al., 2011; Michelson, June, Vives, Testa, & Marchione, 1998; Rufer et al., 2006; Spitzer, Barnow, Freyberger, & Grabe, 2007), while providing a substantial economic burden (see Brand, Lanius, Vermetten, Loewenstein, & Spiegel, 2012, for a review). Research on the potential causes of the poor therapeutic outcome of these patients is thus urgently needed (Lanius, 2015).

A Dissociative Disorder Not Otherwise Specified (DDNOS) Type 1 can be diagnosed if the diagnostic criteria for Dissociative Identity Disorder (DID) are partly present. DDNOS Type 1 is highly prevalent, as the diagnostic criteria for DID are tightly defined (Sar, 2011). Patients that suffer from all of the DID criteria but identity alteration can be diagnosed with DDNOS Type 1a, whereas all DID criteria but amnesia lead to the diagnosis of DDNOS Type 1b (American Psychiatric Association, 1994). The following diagnostic criteria are required to diagnose a DID according to the DSM-5 (American Psychiatric Association, 2013): (A) identity alteration (‘disruption of identity characterized by two or more distinct personality states’ (p. 292) involving ‘marked discontinuity in sense of self and sense of agency, accompanied by related alterations in affect, behavior, consciousness, memory, perception, cognition, and/or sensory-motor functioning’ (p. 292), (B) amnesia (‘recurrent gaps in the recall of everyday events, important personal information, and/or traumatic events that are inconsistent with ordinary forgetting’ (p. 292), (C) distress caused by the disorder, (D) the disturbance not being part of a broadly accepted cultural or religious practice and (E) the absence of substance abuse or another medical condition explaining the symptoms (American Psychiatric Association, 2013).

Several researchers present models to characterize dissociative states. For instance, Frewen and Lanius (2014) distinguish between the normal waking consciousness and trauma-related altered states of consciousness that can be observed especially in people high in dissociative symptomatology. The ‘Window of Tolerance’ model of the effects of complex emotional trauma described by Corrigan, Fisher, and Nutt (2011) postulates that there is a ‘range of optimal arousal states in which emotions can be experienced as tolerable and experience can be integrated’ (p. 17), contrasted by states of sympathetic hyperarousal or parasympathetic hypoarousal. Similarly, van Dijke et al. (2010) found evidence for the existence of three qualitatively distinct experiencing states: over-regulation of affect/inhibitory experiencing states or under-regulation of affect/excitatory experiencing states or a combination of both (see also van Dijke, 2012). Structural dissociation of the personality is defined as a lack of integration between different psychobiological systems constituting personality (Van Der Hart, Nijenhuis, Steele, & Brown, 2004). This concept is based on innate action systems like those described by Panksepp (1998) and on differences between ‘Apparently Normal Part(s) of the Personality’ (responsible for coping with the demands of everyday life) and ‘Emotional Part(s) of the Personality’ (fixated in traumatic memories, which assure survival in situations of severe threat) (Van der Hart, Nijenhuis, & Steele, 2006). In DID research, Reinders et al. (2006) call ‘Apparently Normal Part(s)’ ‘neutral identity states’ and ‘Emotional Parts’ ‘traumatic identity states’.

### 1.2. Links between avoidance and dissociation and treatment of DDNOS and DID

Several researchers have postulated a link between dissociation and avoidance behaviour (Hayes et al., 2004; Hayes, Wilson, Gifford, Follette, & Strosahl, 1996; Van der Hart et al., 2006). According to Hayes et al. (2004, 1996), dissociation belongs to the construct of ‘experiential avoidance’. Experiential avoidance is the ‘phenomenon that occurs when a person is unwilling to remain in contact with particular private experiences (e.g. bodily sensations, emotions, thoughts, memories, behavioral predispositions) and takes steps to alter the form or frequency of these events and the contexts that occasion them’ (Hayes et al., 1996, p. 1154). The concept of structural dissociation of the personality postulates that dissociation is characterized by avoidance and that affected people suffer from a range of phobias, e.g. phobias of different personality parts, phobias of bodily signals and phobias of emotional experience leading to avoidance behaviour (Van Der Hart et al., 2004). Clinical experience has shown that DDNOS/DID patients avoid self-perception, e.g. during personal hygiene or while gazing at their faces in the mirror. This observation has also been described by Frewen et al. (2011) for subjects suffering from PTSD that were asked to look at photos showing their faces.

The Guideline for Treating Dissociative Identity Disorder in Adults by the International Society for the Study of Trauma and Dissociation (ISSTD) proposes a phase-oriented treatment approach for DDNOS and DID (phase 1: establishing safety, stabilization, and symptom reduction; phase 2: confronting, working through, and integrating traumatic memories; phase 3: identity integration and rehabilitation; International Society for the Study of Trauma and Dissociation, 2011). There is a paucity of systematic research on DDNOS/DID treatment and DDNOS/DID treatment outcome, although there is evidence for significant improvement after specific psychotherapeutic treatment (see Brand, Classen, McNary, & Zaveri, 2009, for a review). In those studies reviewed by Brand et al. (2009), specific treatment interventions are not clearly defined. A survey of treatment interventions for DDNOS/DID patients by Brand, Myrick, et al. (2012) describes common treatment techniques mostly matching the ISSTD Guidelines. However, we are not aware of research explicitly focusing on self-perception, especially on the integration of facial mirror-confrontation (CFM) into treatment approaches for DDNOS or DID.

### 1.3. Mirror exposure in post-traumatic disorders/mental disorders

Previous studies have investigated the effect of full-body mirror-confrontation and have reported negative self-referential processing in post-traumatic conditions: Winter, Koplin, and Lis (2015) found a strong trend towards self-awareness avoidance in Borderline Personality Disorder patients and a more intentional choice of avoidance in a full-body mirror exposure paradigm in which those patients and healthy controls (HCs) were asked to choose either a seat facing or not facing a mirror. Furthermore, Borgmann, Kleindienst, Vocks, and Dyer (2014) reported that PTSD patients exhibited more negative emotions and cognitions and higher dissociative states during full-body mirror exposure than HCs. The authors conclude that the patients might perceive their bodies as a trigger for intrusions and emotional suffering (Borgmann et al., 2014). Using a paradigm akin to CFM (asking participants to look at photos of their faces), Frewen et al. (2011) have furthermore reported negative self-referential processing in PTSD patients compared to HCs. Moreover, full-body mirror exposure is used as a therapeutic tool in different psychopathological conditions, e.g. in the treatment of eating disorders (Vossbeck-Elsebusch, Vocks, & Legenbauer, 2013), and might also be helpful in other disorders, such as Body Identity Integrity Disorder (Blom, 2017).

### 1.4. Psychophysiology

#### 1.4.1. Psychophysiology of dissociative stress reactions

There are few studies reporting on the psychophysiology of dissociative stress reactions. Most of these studies did not assess for sympathetic and parasympathetic drive simultaneously. When exposed to a trauma script, 30% of PTSD patients, who usually display psychophysiological hyperarousal under stressful conditions, exhibit blunted autonomic reactivity and are thus classified as ‘physiological non-responders’ (Orr, Metzger, & Pitman, 2002; Orr & Roth, 2000) or ‘dissociative subtype’ of PTSD (Lanius, Bluhm, Lanius, & Pain, 2006; Lanius et al., 2002). Similarly, research by Sierra et al. (2002, 2006) reported on blunted autonomic reactivity (skin conductance level) to unpleasant stimuli and unpleasant emotions in patients affected by Depersonalization-Derealization Disorder. Moreover, Ebner-Priemer et al. (2005) demonstrated a blunted autonomic reactivity in a startle response paradigm (left orbicularis oculi electromyogram) in highly-dissociative Borderline Personality Disorder patients. Schmahl, Elzinga, and Bremner (2002) described a decline in cardiac psychophysiological reactivity during a dissociative reaction in a case study of a Borderline Personality Disorder patient (heart rate, diastolic blood pressure). Sack, Cillien, and Hopper (2012) found an association between acute dissociative stress reactions and an inhibition in psychophysiological arousal (heart rate, parasympathetic cardiac activity reflected by the Root Mean Square of Successive Differences RMSSD) during script-driven trauma imagery in a population of traumatized subjects, partly suffering from PTSD and/or DD. In patients affected by Dissociative Identity Disorder, Reinders et al. (2006) described differences in psychophysiological reactivity (e.g. heart rate, heart rate variability) to a trauma script between ‘neutral identity states’ and ‘traumatic identity states’. ‘Traumatic identity states’ showed a higher heart rate and tendencies towards a lower heart rate variability during the trauma script paradigm than the ‘neutral identity states’. Those differences could not be observed during a neutral script (Reinders et al., 2006).

In the context of life-threatening events (see Schauer & Elbert, 2010, for a review), dissociative reactions have been associated with parasympathetic activity. Lazarus and Folkman (1984) define coping as ‘constantly changing cognitive and behavioral efforts to manage specific external and/or internal demands that are appraised as taxing’ (p. 141). Active coping (‘fight or flight’/’defensive’ response) is associated with an increase in heart rate, sympathetic activation and vagal withdrawal. Passive coping (‘conservation-withdrawal’) on the other hand is characterized by a parasympathetically induced bradycardia with sympathetic coactivation (Bosch, De Geus, Veerman, Hoogstraten, & Nieuw Amerongen, 2003). Bosch et al. (2001, 2003) have investigated psychophysiological reactivity in healthy participants using an active vs. passive coping paradigm. While they observed a rise in heart rate and sympathetic activity and a decline in parasympathetic drive in the active coping mode, the passive coping mode was marked by heightened vagotonus and moderate coactivation of sympathetic and parasympathetic activity. Similar to the psychophysiological hallmarks of ‘passive coping’ (Bosch et al., 2003, 2001), Scaer (2001) and Schore (2001) hypothesized sympathetic and parasympathetic coactivation in dissociative stress reactions.

#### 1.4.2. Psychophysiology and mirror-confrontation

Psychophysiology during full-body but not during solely facial mirror-confrontation has been investigated in some clinical conditions. Studies on highly body-dissatisfied students (Servián-Franco, Moreno-Domínguez, & Del Paso, 2015) and eating disorder patients (Vocks, Legenbauer, Wächter, Wucherer, & Kosfelder, 2007) have shown a blunted autonomic reactivity (heart rate, skin conductance level) in the face of considerable emotional suffering during full-body mirror exposure. Sloan (2004) has focused on the relationship between experiential avoidance and psychophysiology (heart rate). She demonstrated that a more intensive emotional experience correlated with a more blunted heart rate reactivity to emotional stimuli in individuals with high versus low experiential avoidance.

### 1.5. The present study

As DDNOS patients have clinically described their own faces as a particularly rejected part of their body, we assumed that self-perception, operationalized by a CFM paradigm, might be associated with acute stress and dissociation and might counteract self-related avoidance behaviour. We thus hypothesized a significant difference in self-estimated stress (**hypothesis 1a**) and dissociation (**hypothesis 2a**) between DDNOS patients and HCs during the CFM. Furthermore, we hypothesized an immediate increase in self-reported stress (**hypothesis 1b**) and dissociation (**hypothesis 2b**) during mirror-confrontation with their faces in DDNOS patients. Regarding psychophysiological parameters, we hypothesized that the course of psychophysiological activity measured by impedance cardiography would differ significantly between patients and HCs (**hypothesis 3a**). In particular, we assumed an enhanced coactivation of sympathetic and parasympathetic tone during mirror-confrontation in the patient group (PG) in relation to the HCs (**hypothesis 3b**).

## 2. Materials and methods

### 2.1. Participants

We calculated sample sizes using G*Power (Faul F, University of Kiel, G*Power Version 3.1.7). A sample size of 18 participants in each group was determined to have 80% power to detect an assumed medium effect size at a 0.05 two-sided significance level. Eighteen DDNOS patients (17 female) and 18 HCs (17 female) thus participated in the study. Patients in the study were consecutive inpatients and outpatients at a centre specialized in psychotraumatology at the Department of Psychosomatic Medicine and Psychotherapy, Klinikum rechts der Isar, Technical University of Munich, Germany. HCs were recruited from employees and medical students at Klinikum rechts der Isar, Technical University of Munich, without any current mental disorders. The two groups were matched in terms of gender, age and body mass index (BMI). In order to be included in the PG, a DD diagnosis based on the German version of the Mini-SCID-D interview (total score of 10 or more points) was required (Gast, Zündorf, & Hofmann, 1999; Steinberg, Rounsaville, Buchannan, & Chichetti, 1992). The patients suffered from a DDNOS Type 1. All the patients had an additional PTSD due to multiple trauma (*M *= 3.2 traumatizations, *SD *= 1.3), fulfilled the criteria of the dissociative subtype of PTSD according to the DSM-5 and, additionally, had fragmentation symptoms (identity alteration, identity confusion, amnesia). A total of 50% suffered from other common mental disorders. Prospective participants were excluded if they had severe somatic or neurological disorders or took betablockers or benzodiazepines. None of the participants took steroids or antiarrhythmic agents; none of the patients and two HCs took antihypertensive drugs. HCs were free from current psychopharmaceutical treatment, while 44.4% of the patients took antidepressant or neuroleptic drugs. Drugs other than psychopharmaceuticals were taken by 27.8% of the patients and 16.7% of the HCs (levothyroxine: three patients, one HC; non-steroidal antirheumatic drugs: two patients). Exclusion criteria were a current severe depressive episode, a lifetime psychotic disorder or a lifelong history of substance abuse. HCs were excluded if they suffered from a current mental disorder. The study design was approved by the ethics committee of the Technical University of Munich (proposal 1/14 S) and written informed consent was obtained from all patients according to the Declaration of Helsinki. Out of the 60 patients invited to participate in the study, 22 failed to respond or opted not to participate. Another 18 did not meet the study criteria and two were unable to follow instructions during the experiment. HCs were recruited by posting notices in the hospital and on the intranet. From the 200 responses received, 23 HCs were initially selected for matching. Four of these were subsequently excluded due to a current mental disorder, while one was excluded as a result of language difficulties.

### 2.2. Instruments

We used the validated German translations of all instruments employed in this study.

#### 2.2.1. Interviews

The SCID-D interview (Steinberg, 1994) comprises the subscales amnesia, depersonalization, derealization, identity disturbance and identity alteration (Steinberg, 1994). Patients were diagnosed as having DD and PTSD by means of the Mini-SCID-D interview (Gast et al., 1999; Steinberg et al., 1992) (short form of the SCID-D interview) and the SCID-PTSD interview (First, Spitzer, Gibbon, & Williams, 1997; Wittchen, Zaudig, & Fydrich, 1997) according to DSM-IV administered by a trained clinician (ES).

#### 2.2.2. Dissociation and trauma

To evaluate trait dissociation, we used the Dissociative Experiences Scale (Bernstein & Putnam, 1986). The severity of trauma-related symptoms was assessed by administering the Impact of Event Scale (IES; Horowitz, Wilner, & Alvarez, 1979; Hütter & Fischer, 1997). Child abuse and neglect were measured retrospectively with the Childhood Trauma Questionnaire (CTQ) and its five subscales (emotional abuse, physical abuse, sexual abuse, emotional neglect, physical neglect) (Bernstein & Fink, 1998).

#### 2.2.3. General mental strain

We used the Brief Symptom Inventory (BSI) to measure general mental strain (Derogatis & Melisaratos, 1983).

#### 2.2.4. Self-perception, self-regulation and self-compassion

The Acceptance and Action Questionnaire (AAQ; Hayes et al., 2004) was used to assess experiential avoidance. Moreover, we administered the Hannover Self-Regulatory Inventory (HSRI; Jäger, Schmid-Ott, Dölle-Lange, & Sack, 2012) to evaluate self-regulatory capacities and ego functions. To estimate self-compassion, we asked participants to complete the Self-Compassion Scale (SCS; Neff, 2003).

#### 2.2.5. Self-reported in-session stress and dissociation

We used the Subjective Units of Disturbance (SUD) Scale to assess subjective distress on a scale of 0 (‘no distress’) to 10 (‘maximum distress’) (Wolpe, 1969). The Responses to Script-Driven Imagery (RSDI) Scale (Hopper, Frewen, Sack, Lanius, & van der Kolk, 2007) was developed to assess acute intrusion, avoidance and dissociation symptoms during script-driven imagery (imagining stressful traumatic events) and thus stress-inducing paradigms. The RSDI subscale dissociation (RSDI-SSD) consists of four items and was used to assess state dissociation in the present study. Each item can range from 0 to 6 points, which implies a potential total score between 0 and 24 points. A higher RSDI-SSD score means more severe state dissociation experiences.

### 2.3. Procedures

**Figure 1.**
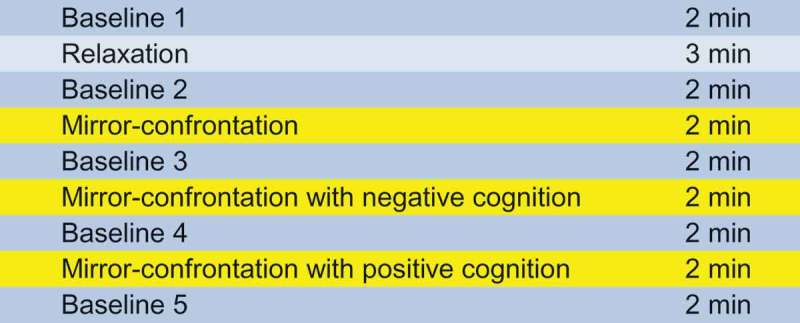
Course of the different measurement phases.

The experimental process is shown in Figure 1. During the experiment, participants sat in a comfortable chair 1 metre from a 40 × 40 cm mirror that reflected their face. There were three mirror-confrontation phases (Figure 1, yellow) of 2 minutes each during which the participants were told to look at their face without any instruction (mirror-confrontation, MC) and, subsequently, to silently think about first a negative (mirror-confrontation with negative cognitive accompaniment, MC neg) and then a positive cognition (mirror-confrontation with positive cognitive accompaniment, MC pos) during CFM. We chose this experimental procedure consisting of three CFM phases (a) to assess the dependent variables following pure mirror-confrontation, (b) to raise the probability for comparability between both groups concerning autonomic nervous system parameters by trying to induce psychological stress also in HCs during mirror-confrontation with negative cognition and (c) to add the third mirror-confrontation with positive cognition at the end of the experiment due to ethical reasons. Participants chose pre-defined negative and positive cognitions from the Eye Movement Desensitization and Reprocessing (EMDR) manual (Shapiro, 2001). They were instructed to describe their most negative cognitions which had to produce a SUD score of at least 7 in order to qualify as an accompanying cognition. It was not possible for HCs to obtain a SUD score of 7 or more as they could not think about themselves so negatively. The three mirror conditions were not permuted in order to avoid carry-over effects (MC neg to MC) and for ethical reasons (MC pos should be at the end of the experiment).

Before, between and after the mirror-confrontation periods, baseline conditions (2 min each, imagining washing the dishes) were performed (Figure 1, blue). Between the first baseline measurement and the first mirror-confrontation phase, the participants undertook a relaxation task (Wengenroth, 2012, p. 143, adapted version) (Figure 1, light blue) to limit anticipatory anxiety and for ethical reasons. Apart from the three mirror-confrontation phases, the mirror was covered. At the end of each mirror-confrontation phase and after the ‘baseline 1’ phase, ‘baseline 5’ phase and the ‘relaxation’ task, SUD and RSDI-SSD were assessed. During the entire experiment, electrocardiography and impedance cardiography data were collected continuously.

### 2.4. Impedance cardiography and Vrije Universiteit Ambulatory Monitoring System

Peripheral psychophysiological activity was assessed with the validated Vrije Universiteit Ambulatory Monitoring System (VU-AMS, Vrije Universiteit, Department of Psychophysiology, Amsterdam, Netherlands) Model 5 FS by recording electrocardiography and impedance cardiography with a sample rate of 1000 Hz (De Geus & van Doornen, 1996; De Geus, Willemsen, Klaver, & van Doornen, 1995; Riese et al., 2003; Willemsen, De Geus, Klaver, van Doornen, & Carroll, 1996). The VU-AMS calculated interbeat intervals (IBIs) to assess the heart period between adjacent R-R signals. The pre-ejection period (PEP) served as a measure of peripheral sympathetic drive and myocardial contractility (Berntson, Lozano, Chen, & Cacioppo, 2004; Cacioppo et al., 1994; Newlin & Levenson, 1979; Schächinger, Weinbacher, Kiss, Ritz, & Langewitz, 2001). PEP was calculated by the VU-AMS adding a fixed Q-R interval of 48 ms to the time from R to the B-point (De Geus & van Doornen, 1996). A shorter PEP means an increase in sympathetic drive (Schächinger et al., 2001). Parasympathetic drive was measured using RMSSD as an index of heart rate variability (Malik et al., 1996). In accordance with the recommendation of the Task Force of the European Society of Cardiology and the North American Society of Pacing and Electrophysiology (Malik et al., 1996), the RMSSD of each heartbeat was calculated from the five preceding and following heart periods. Given the skewed distribution of the RMSSD, the natural logarithm of RMSSD, lnRMSSD, was employed to conduct further analyses (Malik et al., 1996). A rise in lnRMSSD represented an increase in parasympathetic drive. Physiological data were processed using VU-DAMS 3.2 software. Suspicious beats were detected automatically by the VU-AMS. We visually inspected and manually corrected or deleted suspicious beats and artefacts according to the VU-DAMS manual (VU-DAMS, 2013). All files were processed.

### 2.5. Data analysis

For psychophysiological data, we calculated the mean of the first minute of the different phases in order to detect effects directly associated with the introduced experimental condition and used it for further analyses. Statistical analyses were conducted using SPSS version 22.0 applying a statistical threshold of *p* < .05 (two-sided). In the case of nominal data, χ^2^ tests were used. T-tests for independent samples were computed in order to analyse initial differences between patients and HCs in each variable. For the analysis of the dependent variables, changes within each group were analysed using linear mixed models: with the participant as a random effect and the time as a fixed effect (Singer, 1998). Differences between groups were analysed accordingly with group as fixed effect. All mixed models controlled for the initial value of the dependent variable, respectively.

## 3. Results

### 3.1. Clinical, psychometric and demographic sample characteristics

The sample comprised 18 patients and 18 HCs, of which 17 in each group were female. Sociodemographic data and sample characteristics are presented in Table 1. Due to matching, the two experimental groups did not differ significantly in age, share of high school diploma holders, gender and BMI. Patients reported an average of 5.1 (*SD *= 5.0) months of psychosomatic and 2.3 (*SD *= 4.0) months of lifetime psychiatric inpatient treatment. Of the patients, all but one had received outpatient psychotherapy. In contrast, only one of the HCs reported to have received outpatient psychotherapy in the past. Comorbid mental disorders were frequent: Major depression (*n *= 9, 50%) or major depression combined with either an anxiety, obsessive-compulsive or eating disorder (*n *= 6, 33.3%) were frequent comorbidities. HCs were free from current psychiatric diagnoses and psychopharmaceutical treatment, while 44.4% of the patients took antidepressant or neuroleptic drugs. Drugs other than psychopharmaceuticals were taken by 27.8% of the patients and 16.7% of the HCs.

**Table 1.** Sociodemographic data and sample characteristics.

	PG	HCs	Group comparison
	*N* = 18	*N* = 18	*p*
**Age** (*M*; *SD*)	41.7 (8.3)	41.1 (10.0)	.86
**Education** (% high school diploma)	50.0	61.1	.50
**Gender** (% female)	94.4	94.4	1.00
**BMI** (*M*; *SD*)	23.6 (4.1)	24.7 (2.9)	.40

Between-group differences: *t*-tests for independent samples (continuous data)/χ^2^-tests (nominal data). Abbreviations: PG = patient group, HCs = healthy controls, *M* = mean, *SD* = standard deviation, BMI = body mass index; * *p* < .05.

Table 2 shows the psychometric data of the DDNOS patients and the HCs. Mini-SCID-D interviews were conducted to determine the severity of dissociative symptoms. While all HCs had dissociation scores of zero (Table 2), the average score for the PG was 11.39 (*SD *= 1.09) (Table 2). In addition to substantial dissociative symptoms in the Mini-SCID-D, criteria for DDNOS were fulfilled according to clinical expert interviews in the PG. All DDNOS patients suffered from comorbid PTSD according to the SCID-PTSD interview, but DDNOS was the most important inclusion criterion for the study and, as clinical expert interviews revealed, DDNOS was the main diagnosis and main complaint that was most prominent clinically in every patient tested. Moreover, all patients fulfilled the criteria of the DSM-5 dissociative subtype of PTSD. In addition, they suffered from fragmentation symptoms like amnesia, identity alteration and identity disturbance. All patients had experienced multiple traumatizations (*M *= 3.2 traumatizations, *SD *= 1.3). HCs comprised both traumatized and non-traumatized individuals. However, their average childhood traumatization scores were significantly lower than in patients. They exhibited lower intensity of overall psychopathological symptoms (Table 2). In contrast to HCs, DDNOS patients showed more intense dissociative symptoms in the DES, severe PTSD symptoms in the IES, reported high childhood abuse and neglect intensity in the CTQ, a severe general mental strain in the BSI, high experiential avoidance, low self-compassion and tended to have only minimum self-regulation abilities (Table 2).

**Table 2.** Psychometric data.

		PG	HCs	Group comparison
	interview/questionnaire (abbreviation) (range)	*M* (*SD*)	*M* (*SD*)	*p*
**Dissociation**	**Mini-SCID-D total score** (0–15)	11.39 (1.09)	0.00 (0.00)	< .001*
	**Mini-SCID-D amnesia** (0–3)	2.44 (0.98)	0.00 (0.00)	< .001*
	**Mini-SCID-D depersonalization** (0–3)	3.00 (0.00)	0.00 (0.00)	< .001*
	**Mini-SCID-D derealization** (0–3)	2.11 (1.08)	0.00 (0.00)	< .001*
	**Mini-SCID-D identity disturbance** (0–3)	2.33 (0.49)	0.00 (0.00)	< .001*
	**Mini-SCID-D identity alteration** (0–3)	1.50 (0.92)	0.00 (0.00)	< .001*
	**Dissociative Experiences Scale (DES)** (0–100%)	27.86 (9.28)	4.35 (2.79)	< .001*
**Trauma**	**PTSD (diagnosed with SCID-PTSD interview)**	Yes	No	–
	**Impact of Event Scale (IES)** (0–75)	54.28 (11.85)	–	–
	**Childhood Trauma Questionnaire (CTQ) total score** (25–125)	82.50 (15.75)	29.25 (3.25)	< .001*
	**CTQ emotional abuse** (5–25)	18.90 (4.85)	6.20 (1.15)	< .001*
	**CTQ physical abuse** (5–25)	12.90 (4.70)	5.15 (0.40)	< .001*
	**CTQ sexual abuse** (5–25)	16.45 (5.60)	5.00 (0.00)	< .001*
	**CTQ emotional neglect** (5–25)	21.45 (2.30)	7.55 (2.00)	< .001*
	**CTQ physical neglect** (5–25)	12.80 (3.80)	5.30 (0.70)	< .001*
**General mental strain**	**Brief Symptom Inventory (BSI) Global Severity Index (GSI)** (0–4)	2.02 (0.6)	0.19 (0.18)	< .001*
**Self-perception/self-compassion**				
Experiential avoidance	**Acceptance and Action Questionnaire (AAQ)** (9–63)	45.11 (6.72)	22.22 (3.46)	< .001*
Self-compassion	**Self-Compassion Scale (SCS)** (1–5)	2.30 (0.48)	3.63 (0.46)	< .001*
Self-regulation	**Hannover Self-Regulatory Inventory (HSRI)** (5–25)	18.95 (2.69)	8.28 (1.31)	< .001*

Between-group differences: *t*-tests for independent samples. Abbreviations: PG = patient group, HCs = healthy controls, *M* = mean, *SD* = standard deviation, SCID-D = Structural Clinical Interview for DSM-IV Dissociative Disorders, DES = Dissociative Experiences Scale, IES = Impact of Event Scale, CTQ = Childhood Trauma Questionnaire, BSI = Brief Symptom Inventory, GSI = Global Severity Index, AAQ = Acceptance and Action Questionnaire, SCS = Self-Compassion Scale, HSRI = Hannover Self-Regulatory Inventory, * *p* < .05.

### 3.2. Self-reported stress and dissociation during CFM

**Figure 2.**
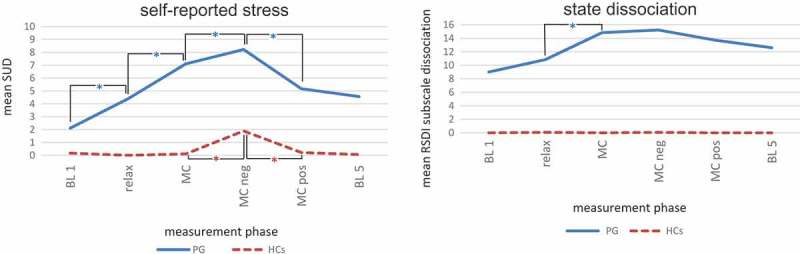
Within-group courses of self-reported stress (Subjective Units of Disturbance, SUD) and self-reported dissociation (Responses to Script-Driven Imagery Scale subscale dissociation, RSDI subscale dissociation) for patients (PG) and healthy controls (HCs). Statistical details of between-group differences are shown in Table 3. Within-group differences: linear mixed models. Abbreviations: SUD = Subjective Units of Disturbance (range: 0–10), RSDI = Responses to Script-Driven Imagery Scale (subscale dissociation: range 0–24), BL = baseline; relax = relaxation, MC = mirror-confrontation, neg = negative (with negative cognitive accompaniment), pos = positive (with positive cognitive accompaniment), PG = patient group, HCs = healthy controls; * *p* < .05.

We assessed the subjective stress response of patients and HCs in the course of the mirror paradigm using the SUD Scale and the RSDI-SSD. The initial between-group differences in baseline SUD levels (T(df) = T(17.00) = 3.90; *p *= .001) and baseline RSDI-SSD levels (T(df) = T(17.00) = 8.72; *p *< .001) were significant. Table 3 shows the mean SUD and RSDI-SSD scores, standard deviations and the mean differences from phase to phase as well as the between-group differences in SUD and RSDI-SSD that were controlled for the baseline SUD/RSDI-SSD levels. Figure 2 indicates SUD and RSDI-SSD experimental progressions over the different time points of the assessment, with significant changes between consecutive conditions within each group indicated with an asterisk. Controlling for the initial values of the dependent variable, linear mixed models yielded significant between-group differences for all measurement periods both for SUD and for RSDI-SSD (Table 3). As expected, we observed a significant increase in self-reported stress and self-reported acute dissociation going from the relaxation task to the neutral mirror condition in the PG (Figure 2, Table 3). Likewise, self-reported stress continued to increase significantly in the PG, going from the neutral to the negative mirror condition (Figure 2, Table 3). Finally, it dropped significantly going from the negative to the positive mirror condition in the PG (Figure 2, Table 3). In the PG, RSDI-SSD showed no within-group differences depending on the cognition (Figure 2, Table 3). For the HCs, the only significant differences in the course of the experiment were a rise of SUD from the neutral to the negative mirror condition and a decline of SUD from the negative to the positive mirror condition (Figure 2, Table 3). The neutral mirror condition did not result in a substantial increase of SUD for the HCs. The HCs did not exhibit state dissociation (RSDI-SSD) over the entire course of the experiment (Figure 2, Table 3).

**Table 3.** Within-group courses of self-reported stress (Subjective Units of Disturbance, SUD) and self-reported dissociation (Responses to Script-Driven Imagery Scale subscale dissociation, RSDI subscale dissociation) for patients (PG) and healthy controls (HCs) (left, also shown in Figure 2) and between-group differences for these parameters (right).

**SUD**	PG			HCs			PG compared to HCs^3^	
	*M* (*SD*)	F^1^	*p*^1^	*M* (*SD*)	F^1^	*p*^1^	F	*p*
BL 1	2.11 (2.00)			0.17 (0.71)				
Relax	4.39 (2.50)	9.12	.005*	0.00 (0.00)	1	.331	39.93	< .001*
MC	7.11 (1.78)	21.07	< .001*	0.11 (0.32)	2.13	.163	189.93	< .001*
MC neg	8.22 (1.70)	10.00	.006*	1.89 (1.41)	27.82	< .001*	90.15	< .001*
MC pos	5.17 (2.43)	34.44	< .001*	0.22 (0.55)	28.33	< .001*	42.95	< .001*
BL 5	4.56 (2.75)	1.27	.276	0.06 (0.24)	0.86	.434	28.14	< .001*
**RSDI-SSD**	PG			HCs			PG compared to HCs^3^	
	*M* (*SD*)	F^1^	*p*^1^	*M* (*SD*)^2^	–	–	F	*p*
BL 1	9.00 (4.38)			0.00 (0.00)	–	–		
Relax	10.83 (5.56)	2.87	.109	0.06 (0.24)	–	–	4.29	.046*
MC	14.83 (4.06)	6.08	.019*	0.00 (0.00)	–	–	52.57	< .001*
MC neg	15.22 (5.51)	0.11	.749	0.06 (0.24)	–	–	34.19	< .001*
MC pos	13.72 (6.06)	1.18	.292	0.00 (0.00)	–	–	16.15	< .001*
BL 5	12.61 (5.49)	0.51	.483	0.00 (0.00)	–	–	8.33	.007*

^1^ Mean within-group difference related to precedent measuring period calculated using linear mixed models.

^2^ Statistical analysis meaningless due to lack of variation.

^3^ Between-group differences: linear mixed models.

Abbreviations: *M* = mean, *SD* = standard deviation, PG = patient group, HCs = healthy controls, SUD = Subjective Units of Disturbance (range: 0–10), RSDI-SSD = Responses to Script-Driven Imagery Scale subscale dissociation (range: 0–24), BL = baseline, Relax = relaxation, MC = mirror-confrontation, neg = negative (with negative cognitive accompaniment), pos = positive (with positive cognitive accompaniment), * *p* < .05.

Two patients stopped before the end of the experiment; they reported very severe stress and dissociative symptoms and were thus not able to follow the instructions anymore. Five patients reported that disturbing memories arose during the CFM exposure. Five patients felt extreme aggressiveness upon looking at their faces in the mirror; they reported that they had an urge to break the mirror and hurt themselves. All participants talked to the investigator after the experiment for about 10 minutes to debrief and ensure remission of these stress-elicited symptoms. Six participants had to be treated by an acute crisis intervention (25–50 min) following the experiment to improve grounding ability, emotion regulation and to ensure prevention of the onset of auto-aggressive symptoms. If they were not currently engaged in psychotherapy that warranted sufficient follow-up, one or more follow-up psychotherapy session/s was/were arranged. In contrast, the HCs did not manifest any of the aforementioned phenomena but remained rather unconcerned by the experimental procedure.

### 3.3. Sympathetic and parasympathetic tone during CFM assessed by impedance cardiography

**Figure 3.**
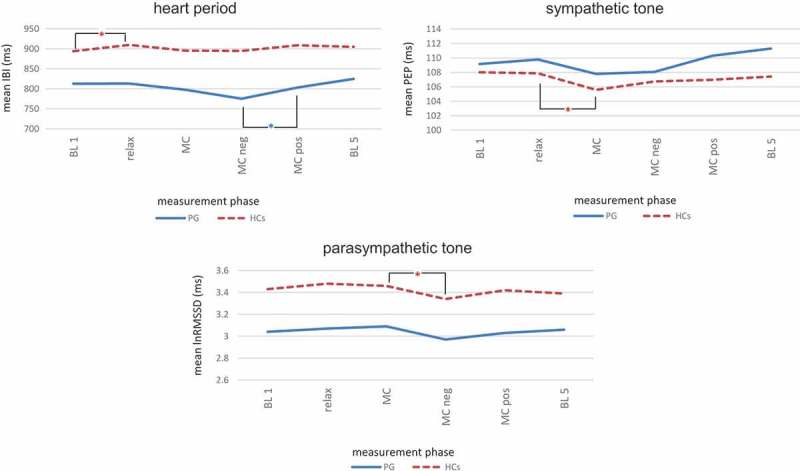
Within-group courses of heart period (inter-beat interval, IBI), sympathetic tone (pre-ejection period, PEP) and parasympathetic tone (natural logarithm of the Root Mean Square of Successive Differences, lnRMSSD) in patients (PG) and healthy controls (HCs). Statistical details of between-group differences are depicted in Table 4. Within-group differences: linear mixed models. Abbreviations: IBI = interbeat interval, PEP = pre-ejection period, lnRMSSD = natural logarithm of Root Mean Square of Successive Differences, BL = baseline; relax = relaxation, MC = mirror-confrontation, neg = negative (with negative cognitive accompaniment), pos = positive (with positive cognitive accompaniment), PG = patient group, HCs = healthy controls; *p < .05.

The between-group differences in baseline IBI (heart period) and lnRMSSD (parasympathetic tone) levels were significant (T(df) = T(34,0) = −2.02; *p* = .05), whereas the baseline PEP (sympathetic drive) levels did not differ (T(df) = T(34,0) = −0.19; *p* = .85). Table 4 indicates IBI, PEP and lnRMSSD means, standard deviations and mean differences from period to period within each group calculated using linear mixed models, as well as the between-group differences for each measurement period calculated by means of linear mixed models. Figure 3 shows the intra-group changes in IBI, PEP and lnRMSSD from one measuring period to the next, again with significant changes between consecutive conditions within each group indicated with an asterisk. The three parameters followed similar tracks for the PG and the HCs, and there were no significant differences between the PG and the HCs when controlling for the initial value of the dependent variable (Figure 3, Table 4). Throughout the entire experiment, the patients had a lower IBI indicating a higher heart rate, as well as a higher PEP indicating lower sympathetic drive and a lower lnRMSSD representing a lower parasympathetic tone (Figure 3, Table 4). For both the PG and the HCs, there were only few significant within-group changes (Figure 3, Table 4), e.g. a significant decline in lnRMSSD in the direction of the mirror-confrontation accompanied by a negative cognition in the HCs.

**Table 4.** Within-group courses of heart period (inter-beat interval, IBI), sympathetic tone (pre-ejection period, PEP) and parasympathetic tone (natural logarithm of the root mean square of successive differences, lnRMSSD) for patients (PG) and healthy controls (HCs) (left, also shown in Figure 3) and between-group differences (right).

	PG			HCs			PG compared to HCs^2^	
	*M* (*SD*)	F^1^	*p*^1^	*M* (*SD*)	F^1^	*p*^1^	F	*p*
**IBI (ms)**								
BL 1	812.63 (96.81)			894.03 (138.92)				
Relax	813.07 (94.50)	0.01	.927	909.75 (144.83)	4.44	.050*	2.68	.111
MC	797.58 (89.71)	2.78	.114	895.53 (141.81)	3.69	.072	2.40	.131
MC neg	775.32 (90.44)	3.62	.074	894.57 (163.75)	0.01	.941	3.77	.061
MC pos	803.39 (112.08)	5.16	.036*	908.64 (143.80)	1.97	.179	2.01	.165
BL 5	824.82 (111.91)	1.21	.311	904.79 (133.92)	0.71	.618	0.02	.885
**PEP (ms)**								
BL 1	109.17 (17.92)			108.02 (18.28)				
Relax	109.78 (18.82)	0.34	.568	107.88 (18.39)	0.02	.89	0.27	.608
MC	107.79 (18.87)	1.09	.311	105.58 (16.71)	6.55	.020*	0.39	.539
MC neg	108.07 (18.44)	0.03	.864	106.74 (18.58)	0.95	.343	0.01	.916
MC pos	110.32 (20.49)	3.43	.082	106.98 (18.25)	0.09	.763	0.83	.369
BL 5	111.30 (18.34)	0.70	.625	107.43 (17.93)	1.40	.233	1.89	.179
**lnRMSSD (ms)**								
BL 1	3.04 (0.54)			3.43 (0.60)				
Relax	3.07 (0.44)	0.20	.665	3.48 (0.63)	0.53	.475	0.70	.407
MC	3.09 (0.44)	0.24	.633	3.46 (0.66)	0.08	.776	0.08	.777
MC neg	2.97 (0.52)	3.03	.1	3.34 (0.66)	4.92	.040*	0.05	.829
MC pos	3.03 (0.52)	0.88	.36	3.42 (0.69)	1.76	.202	0.07	.796
BL 5	3.06 (0.58)	0.16	.976	3.39 (0.62)	0.51	.77	0.001	.972

^1^ Mean within-group difference related to precedent measuring period calculated using linear mixed models.

^2^ Between-group differences: linear mixed models.

Abbreviations: *M* = mean, *SD* = standard deviation, PG = patient group, HCs = healthy controls, IBI = interbeat interval, PEP = pre-ejection period, lnRMSSD = natural logarithm of Root Mean Square of Successive Differences, MC = mirror-confrontation, neg = negative (with negative cognitive accompaniment), pos = positive (with positive cognitive accompaniment), BL = baseline, Relax = relaxation, * *p* < .05.

## 4. Discussion

We compared the within- and between-group differences of self-reported stress (SUD) and dissociation (RSDI-SSD) in DDNOS patients versus HCs in a CFM paradigm and simultaneously explored potential between-group and within-group differences in psychophysiological activity. Upon CFM exposure, we observed a striking discrepancy between considerable self-reported stress and dissociation and a blunted sympathetic and parasympathetic reactivity in DDNOS patients that was not present in HCs.

### 4.1. Self-reported stress and dissociation

In line with our hypotheses, we found significant immediate increases in self-reported stress assessed by SUD (**hypothesis 1b**) and in acute dissociation represented by RSDI-SSD (**hypothesis 2b**) for the patients when exposing them to the neutral mirror condition (Figure 2, Table 3). These increases were not present in the HCs. In all measurement phases, SUD and RSDI-SSD of the PG were significantly higher than in the HCs (**hypothesis 1a and 2a**).

To our knowledge, there is no research reporting on the potential distress-inducing effect of explicit facial mirror-confrontation in mental disorders. That is why our data are discussed in relation to previous research focusing on full-body mirror-confrontation. Investigations on the effect of full-body mirror-confrontation showed negative emotions and cognitions in highly body-dissatisfied individuals (Servián-Franco et al., 2015), in eating disorder patients (Vocks et al., 2007) and in PTSD patients (Borgmann et al., 2014). The results of the present study fit with the data by Winter et al. (2015) reporting on full-body mirror avoidance in Borderline Personality Disorder patients, are in keeping with the concept of experiential avoidance (Hayes et al., 2004, 1996) and are in line with the Theory of Structural Dissociation of the Personality by van der Hart et al. (2006) describing self-perception phobia. Furthermore, our results are in keeping with the observations by Frewen et al. (2011) who found negative self-referential processing in PTSD patients when looking at photos of their faces, a paradigm similar to our CFM paradigm. However, Frewen et al. (2011) did not report if a DD diagnosis was present, so it remains unclear whether their observations could be linked to PTSD, to dissociation, or both. Additionally, they investigated a dependent variable different from the one studied here (self-referential processing vs. stress/dissociation in our paradigm) and, moreover, did not assess psychophysiological data. The finding of a high level of dissociative symptoms during mirror exposure in PTSD patients by Borgmann et al. (2014) is also in line with our findings. However, these colleagues employed full-body mirror-confrontation and not, as in our study, explicit facial mirror-confrontation.

While their results can be associated with a negative body image, our findings might provide information about a construct that could be called ‘mind image’. After having finished the experiments, several DDNOS patients spontaneously reported that their biggest problem was not their external appearance, but their character traits and subjective feelings like feeling bad, guilty or unworthy. Interestingly, most of those patients who chose not to participate felt that looking at their faces in the mirror three times for two minutes each would be unbearable, implying considerable avoidance of seeing their faces. Whereas Borgmann et al. (2014) assumed that the own body might trigger intrusions and emotional suffering in PTSD patients, our data suggest that their own face, independent from other parts of the body, might constitute a trigger for DDNOS patients with comorbid PTSD.

### 4.2. Psychophysiology

The patients showed lower IBI, higher PEP and lower lnRMSSD and thus an elevated heart rate and a lower sympathetic and parasympathetic tone when compared to the HCs (Figure 3, Table 4). For heart period (IBI) and parasympathetic drive (lnRMSSD), initial differences between both groups were significant. Regarding **hypothesis 3a**, heart period (IBI), sympathetic drive (PEP) and parasympathetic drive (lnRMSSD) did not differ significantly between both groups during the experiment when controlling for the initial value of the dependent variable (Table 4). In the PG, there were slight within-group changes for IBI, PEP and lnRMSSD depending on the cognition during CFM that, with one exception (significant increase in IBI and thus drop in heart rate from MC neg to MC pos), were not statistically significant (Figure 3, Table 4). Interestingly, in the HCs, mirror-confrontation combined with simultaneous focus on a negative cognition about themselves led to a significant decline in lnRMSSD, which accompanied a significant increase in self-reported stress represented by SUD. In contrast to **hypothesis 3b**, we did not observe a coactivation of sympathetic and parasympathetic tone associated with the potential stress reaction during CFM in the PG (Figure 3, Table 4).

While there was thus a discrepancy between high self-reported stress/dissociation and blunted autonomic reactivity in the PG, a concordance between self-reported stress/dissociation and autonomic reactivity was present in the HCs. Self-reported stress for the evaluation of the negative cognition was much less intense for the HCs than in the PG (maximum SUD of 3 in the HCs vs. minimum SUD of 7 in the PG). In this respect, the discrepancy between a considerably higher stress level and the observed blunted autonomic nervous system reactivity in the PG appears even more striking. HCs displayed no self-reported stress/dissociation upon confrontation with the neutral mirror condition and, accordingly, did not exhibit any changes in sympathetic and parasympathetic drive. The absence of between-group differences for IBI, PEP and lnRMSSD (Table 4) thus may not necessarily signify a similarity in the autonomous nervous system reactions between the PG and the HCs, but instead may result from a blunted sympathetic and parasympathetic reactivity in DDNOS patients and from an absence of distinct stress in the HCs. This speculation is strongly supported by a study of Zaba et al. (2015) who demonstrated that PTSD patients with a blunted response of another major stress hormone system, the hypothalamic-pituitary-adrenal (HPA) axis, exhibited a higher prevalence of trauma-related dissociative symptoms in comparison to PTSD patients showing an almost undisturbed HPA axis response. Thus, dissociative symptoms might in principal be associated with a blunting of endocrine stress responses.

Cardiac psychophysiological reactions using impedance cardiography in DDNOS patients during CFM have not yet been investigated. We observed significant self-reported stress and acute dissociation reactions in DDNOS patients. Consequently, our data are discussed here in the context of literature on the psychophysiology of dissociative stress reactions in general. Our observations are in line with the observation of ‘physiological non-responders’ in PTSD patients (30% of PTSD patients) (Orr et al., 2002; Orr & Roth, 2000) and the ‘dissociative subtype’ of PTSD (30% of PTSD patients) (Lanius et al., 2006, 2002) showing a blunted heart rate reaction in a trauma script paradigm. Furthermore, our results extend previous work by Sack et al. (2012) reporting a positive correlation between acute dissociative symptoms and a reduced psychophysiological arousal. Moreover, our data are in accordance with ‘autonomic blunting’ in patients suffering from Depersonalization-Derealization Disorder (Sierra et al., 2002; Sierra, Senior, Phillips, & David, 2006). This is not surprising, as all of our study patients were diagnosed with depersonalization and/or derealization (Table 2). However, a novel finding in our study is that autonomic blunting during CFM is also present in patients additionally suffering from fragmentation symptoms. Besides research on Depersonalization-Derealization Disorder and on PTSD, some investigations on the psychophysiology of Borderline Personality Disorder and DID under stressful conditions can be related to our results. Research on Borderline Personality Disorder patients demonstrated autonomic blunting associated with state dissociation (Ebner-Priemer et al., 2005; Schmahl et al., 2002) and is thus in line with our observations. Our psychophysiological data are furthermore in agreement with the blunted psychophysiological reactions of DID patients in their ‘neutral identity state’ when exposed to a trauma script (Reinders et al., 2006). Although none of our study participants completely fulfilled the diagnostic criteria for DID, they exhibited amnesia or identity alteration in terms of a DDNOS Type 1, but not both of these symptoms, as is required for a DID diagnosis. Assuming that in DDNOS, an ‘Apparently Normal Part of the Personality’/‘neutral identity state’ and ‘Emotional Parts of the Personality’/’traumatic identity states’ are present just like they are in DID (Van der Hart et al., 2006), our PG might have been in their ‘Apparently Normal Part of the Personality’/’neutral identity state’ and possibly not in one of their ‘Emotional Parts of the Personality’/’traumatic identity states’ concerning their psychophysiological reactions.

Some previous studies focused on distress and psychophysiological reactivity during full-body, but not during explicit facial mirror exposure. Research on highly body-dissatisfied participants (Servián-Franco et al., 2015) and on eating disorder patients (Vocks et al., 2007) revealed a striking discrepancy between high subjective distress and a blunted autonomic reactivity during full-body mirror exposure and is thus to some extent in line with our observations. Clandestine dissociative comorbidity might be a possible explanation for those previous observations. However, those authors investigated full-body and not solely facial mirror-confrontation as we did. In addition, our data are in keeping with Sloan (2004) who reported the occurrence of blunted autonomic reactivity despite elevation in emotional reactivity in participants suffering from intense experiential avoidance. As our DDNOS sample showed high scores in the AAQ, a measure for experiential avoidance, the observed blunted autonomic reactivity in our patient sample might be associated with high experiential avoidance.

On the other hand, our impedance cardiography data are not consistent with the theoretical assumptions by Scaer (2001) and Schore (2001). Those authors postulate a sympathetic and parasympathetic coactivation in dissociative stress reactions. In contrast, we observed no substantial autonomic changes in the face of considerable self-reported stress reactions in DDNOS patients during the CFM paradigm. We thus did not observe the expected ‘passive coping’ reaction (Bosch et al., 2003, 2001). In contrast, in the HCs, the small autonomic changes could probably be attributed to insufficient stress levels, rather than to impaired autonomic adaptability. The significant reduction in lnRMSSD during CFM when simultaneously thinking about the negative cognition in the HCs is in line with the results of a meta-analysis by Brindle, Ginty, Phillips, and Carroll (2014) who found a significant decline in parasympathetic drive under stressful conditions in healthy subjects and might signify well-developed autonomic adaptability/flexibility in the HCs.

Our data show a tendency towards a pathologically rigid autonomic regulation capacity in the presence of considerable self-reported stress during CFM in DDNOS patients. We suggest that this autonomic hypoactivity and hyporeactivity can be interpreted as impaired emotion regulation (Thayer, Åhs, Fredrikson, Sollers, & Wager, 2012) and cognitive control (Gillie & Thayer, 2014) capacity during stress-inducing CFM. The observation of autonomic blunting in the face of considerable self-reported stress might be interpreted as an attempt to psychophysiologically remain within the ‘Window of Tolerance’ (Corrigan et al., 2011), eventually due to over-regulation of affect (van Dijke, 2012; van Dijke et al., 2010). It would be interesting to readminister our experiment in a fMRI scanner to explore whether autonomic blunting in our stress paradigm is accompanied by corticolimbic inhibition, a well-known pathomechanism in Depersonalization-Derealization Disorder (Sierra & Berrios, 1998).

### 4.3. Clinical implications

Our results indicate that self-perception constitutes a major stressor for DDNOS patients. This has several clinical implications. Situations or therapeutic interventions enhancing self-perception might lead to high stress levels or crisis situations requiring crisis interventions. It might thus be helpful for therapists as well as DDNOS patients to be aware of this for a good planning of psychotherapy. Furthermore, CFM might be used as a diagnostic tool to find out about the aversiveness of self-perception in DDNOS patients. CFM might also be considered as a therapeutic technique. As the own face potentially constitutes an avoided trigger for traumatic memories in DDNOS patients, CFM might be useful when aiming at confronting the patient with traumatic memories linked to dysfunctional self-perception, in order to overcome self-perception-related avoidance behaviour. However, this would require utmost caution, as CFM induces stress and dissociation. It might thus be advisable to start with short time intervals of CFM and to combine CFM with techniques promoting grounding abilities and mindfulness or self-compassion. Our patients had significantly lower self-compassion scores on the SCS than the healthy participants. The higher self-compassion level in the HCs might also be reflected by the fact that, contrary to the patients, the HCs could not retrieve a negative cognition of a ‘Subjective Units of Disturbance’ level of at least 7 out of 10 (i.e. a highly disturbing negative cognition) when defining a negative cognition about themselves. This might imply that people free from mental disorders possibly manage to overcome stress related to self-perception by a steady feeling of safety concerning the self. Psychotherapeutic approaches promoting self-compassion (e.g. Mindful Self-Compassion; Neff & Germer, 2013) might be helpful for DDNOS patients to cope with the aversiveness of self-perception. Moreover, body therapies such as sensori-motor psychotherapy (Ogden, Minton, & Pain, 2006) or body-mentalization (Spaans, Veselka, Luyten, & Bühring, 2009) might enable DDNOS patients to establish feelings of safety concerning the self and might be useful to overcome self-related avoidance in DDNOS patients. These approaches might help to improve the therapy outcome of this severely disabled patient group.

### 4.4. Limitations

The first limitation concerns the relatively small sample size. Replicating the results with a larger sample could eventually confirm our preliminary results reported here, especially in impedance cardiography (Quintana, Alvares, & Heathers, 2016). Including a single male in each group might moreover be considered a limitation. However, the single male in each group did not vary in any important way from the other participants of the group. DDNOS patients typically share a high level of different mental comorbidities (Freyberger & Spitzer, 2005), resulting in a highly heterogenous patient sample. Concerning medication, we excluded patients who took drugs that have a strong cardiovascular effect (betablockers, benzodiazepines). None of the participants took steroids or antiarrhythmic agents and none of the patients took antihypertensive drugs. However, patients who took antidepressants or neuroleptics and HCs that took antihypertensive drugs other than betablockers were included. Therefore, it cannot be ruled out that medication might have influenced our results (Lanius, 2010), although no participant took tricyclic antidepressants or clozapine, the only psychotropic drugs proven to have a statistically significant impact on heart rate variability (Alvares, Quintana, Hickie, & Guastella, 2016). Conducting a relaxation task at the beginning of the experimental procedure might have influenced our results, too, as it led to substantial stress in the PG. Our HC sample might not have been representative for the average population since it consisted of hospital employees and medical students, who might not represent the average population. Furthermore, excluding for lifelong substance abuse might have confounded our results. Based on our data it is not possible to conclude whether the observed effects are specific to DDNOS patients or are a general sign of psychopathology. Research including clinical control groups like patients with somatic symptom disorders could fill this gap. Moreover, control conditions, e.g. a task in which participants look at faces of other people and/or at other, more neutral stimuli, would have helped to tell whether the found effects are specific to perception of participants’ own faces in the mirror. Furthermore, we did not correct for α error when applying multiple testing, due to the small sample size and the pilot character of the study. Moreover, the chosen interval of 1 min duration for impedance cardiography data analysis of the different measuring periods may have influenced the results. However, we decided to choose the first minute in order to detect effects directly associated with the introduced experimental condition. In addition, we did not control for exercise level and caffeine or alcohol intake, parameters that might have influenced cardiovascular reactivity (Laborde, Mosley, & Thayer, 2017). Also, we did not systematically control gaze direction, although we watched participants and required them to look in the mirror when their gaze wandered. This limitation could be ruled out using cameras or eye tracking.

## 5. Conclusions

We observed a striking discrepancy between highly elevated self-reported stress and dissociation levels and autonomic blunting in the sympathetic and parasympathetic tone in DDNOS patients during a CFM paradigm. Our data provide the first empirical evidence for the assumption that dissociation is associated with a remarkable avoidance of self-perception and that their own face might be considered a trigger for traumatic memories in highly-dissociative patients. Psychotherapeutic approaches promoting self-perception, self-compassion and a feeling of safety concerning the self might be useful to overcome self-related avoidance in DDNOS patients.
